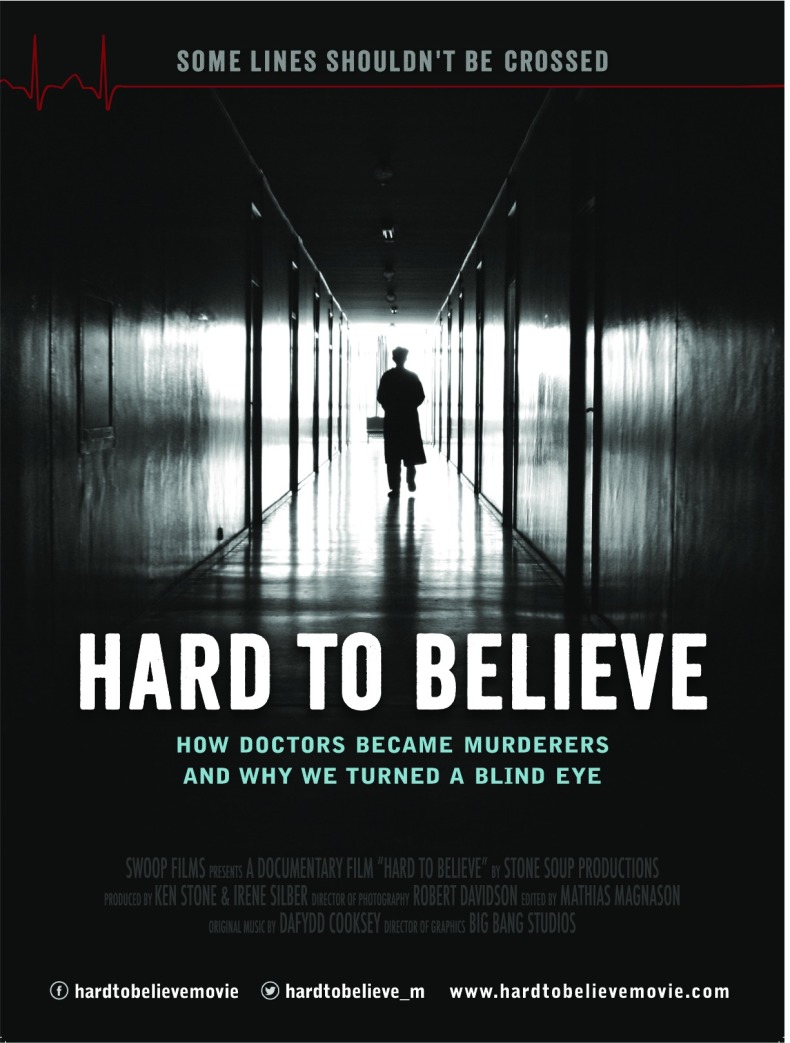# *Hard to Believe*: Produced by Ken Stone and Irene Silber, 2015, Swoop Films and Stone Soup Productions (New York, 56 minutes, unrated)

**DOI:** 10.1007/s11673-016-9717-1

**Published:** 2016-03-30

**Authors:** Holly Louise Northam

**Affiliations:** University of Canberra, Canberra, ACT AUSTRALIA

**Keywords:** Organ trade, Sale, Trafficking, Organ donation, Ethics, China, Human rights, Transplant tourism, Suffering, Consent

## Abstract

This article presents a review of *Hard to Believe*, a compelling documentary reporting the forced organ procurement and death of Chinese prisoners of conscience. The documentary is targeted to ignite political and public pressure to stop these practices that are thought to be motivated by financial and political gain. Narrated by journalist and author Ethan Gutmann, the documentary pricks at the collective conscience, as credible witnesses provide evidence that point to an abrogation of every ethical principle ascribed to legitimate organ procurement.

The title of this documentary, *Hard to Believe*, caught my attention. I felt impelled to watch, conscious of my professional responsibility to be informed, concerned about the implications. Few would be unaware of ongoing protests by Falun Gong practitioners against Chinese human rights abuses. Recently, Chinese officials have acknowledged the use of executed prisoners’ organs for transplantation and have promised a more ethical system of organ transplantation, new regulations, and a plan to stop using prisoners’ organs. Despite this, desperation drives some patients to source organs from illegal market networks, with indisputable evidence that this trade results in human misery.

The credentials of the interviewed experts are impeccable. Narrator Ethan Gutmann, an award-winning human rights investigative journalist and author of *The Slaughter*, is supported by internationally recognized Canadian human rights lawyer David Matas; Professor Arthur Caplan, director of medical ethics at the University of New York; Dr. Enver Tohti, former surgeon from Xinjiang, China; U.S. Republican Congressman Dana Rohrabacher; Dr. Jacob Lavee, president of the Israel Society of Transplantation; Professor Katrina Bramstedt, ethicist at Bond University and an associate editor of the *Journal of Bioethical Inquiry*; and others.

The documentary commences with footage of Falun Gong practitioners outside the fourteenth International Organ Donation Congress in San Francisco. The narrator sets the scene, describing how protesters “felt the weight of many bodies on their shoulders.” The narrative proceeds to highlight the desperate plight of patients awaiting transplantation and to systematically outline the veracity and magnitude of human rights abuses involving forced organ procurement of Chinese minorities, including the Falun Gong, Uyghurs, Tibetans, and “House” Christians. Gutmann argues that a lack of transparency regarding the provenance of tens of thousands of organs transplanted in China is sinister, given that China is the second-largest organ transplanter in the world and reports low rates of voluntary donation. Gutmann and others argue that organs from executed prisoners are purchased by foreigners as well as by wealthy, influential Chinese.

In this documentary, David Matas, who investigates abuse claims by Falun Gong practitioners, reports that following the Chinese crackdown and detention of Falun Gong practitioners in 1999, many thousands of unidentified prisoners of conscience were incarcerated in labour camps and disappeared without trace. Matas and Gutmann describe accounts from many Falun Gong who had been detained and imprisoned and who explained that they and others refused to reveal their names to authorities because they wished to protect their families from a similar interrogation and incarceration.

Matas reveals that the Falun Gong were consistent in describing their experiences of brutality but, unexpectedly, also reported organ “health checks” that involved the collection of large samples of blood at three monthly intervals and unusual eye examinations that did not seem consistent with standard health examinations. According to Matas, the most “chilling thing” to him was that the blood testing and organ and eye exams appeared confined to detainees who were Falun Gong practitioners and that Falun Gong and minority group detainees from diverse locations and circumstances independently reported being involved in similar tests. These incidental findings seem inexplicable to Matas and Gutmann, who do not believe the “health” examinations were motivated by consideration of the detainee prisoners’ best interests.

Gutmann and Matas form the view that it was possible that detainees’ organs were being assessed and used for transplantation based on the compounding evidence from these reports; the significant increase in transplantation rates in China after the Falun Gong persecution commenced; and speculation regarding forced organ removal that arose from detainee witness reports that executed prisoners’ bodies are cremated before their families are notified of the death or have seen the body of the deceased. A breakthrough occurred in Matas and Gutmann’s investigations when a doctor confessed his role in the removal of organs from an executed prisoner.

Dr. Enver Tohti, a former surgeon from Xinjiang, China, also is interviewed about his involvement in the removal of organs prior to a state-sanctioned death. He describes how he and his surgical team were co-opted by a senior doctor to gather surgical equipment, without explanation. They accompanied a supervisor to a site where a planned execution was under way. Dr. Tohti describes how he and his team were pressured again to remove the liver and kidneys of a prisoner who had been shot but who showed signs of life until the organs were excised.

Gutmann argues that this practice is not isolated and that Falun Gong practitioners are specifically targeted for forced organ harvest because their organs are preferred for people purchasing organs. This is because Falun Gong are required to maintain healthy lifestyles and do not smoke or consume alcohol. This argument is supported in the documentary by evidence collected from recorded telephone calls to more than 100 Chinese hospitals, during which doctors assure callers that scheduled transplantation surgery will be conducted using organs specifically chosen from healthy Falun Gong prisoners. The documentary claims that the recorded discussions are between hospital staff and family members of people in need of an organ transplant, who prior to the transplant surgery seek assurances about the quality of organs they are purchasing. The narrator disputes a statement from Chinese authorities that the calls are a hoax, arguing that hundreds of witness statements support the veracity of the recordings. Evidence that transplant tourists are offered short waiting times for scheduled organ transplants and receive young, healthy organs from executed prisoners adds weight to the suspicions. Gutmann argues that the practice described by Dr Tohti of removing organs from dying “executed” prisoners may be motivated by a desire to improve the function of the transplant.

Dr. Jacob Lavee, president of Israel’s Transplantation Society, describes how he was previously complacent when patients returned to Israel with a purchased kidney, believing the donor benefited from the organ sale. In this documentary, Lavee describes how his attitude changed dramatically when a patient told him he was travelling to China for a scheduled heart transplant. This idea was shocking to Lavee, because the circumstances of death that allow heart donation cannot be predicted. Lavee was even more distressed when the scheduled heart transplant went ahead as planned and he found that the patient had all his medical costs covered by insurance. Lavee describes that this information was a “game changer” for him. It led him to initiate the introduction of a landmark law that has significantly reduced Israeli transplant tourism. The law prohibits medical insurers from covering the costs of transplantation for Israelis who receive illegally procured organs bought outside of Israel. Interviewees recount that Spain also has changed its legislation, and although not stated, it is presumably to follow Israel’s lead. Those in the film strongly recommend that similar laws be introduced to reduce the organ trade in the United States, Canada, and other countries with populations known to participate in transplant tourism. It is reported in the documentary that Australia has intervened to minimize the harm involved in these practices by ceasing training of Chinese transplant surgeons, while Malaysia has sought to limit the trade by refusing to fund anti-rejection drugs for patients returning with an organ from China.

Gutmann reports that these international efforts to limit the illegal organ trade were barely noticed in the United States. He argues that it is possibly because the Falun Gong use images that are culturally challenging and may alienate American observers. In the documentary, Falun Gong practitioners are interviewed about their experience of being tortured as detainees. One, “Annie,” speaks of being X-rayed, having her blood tested, and receiving eye and kidney checks. Footage is shown of Falun Gong engaging in a continuous vigil outside the Chinese embassy in London since 2002 in an attempt to raise public awareness of human rights abuses. As Gutmann asks, why is such evil ignored?

Enquiries by European governments and the World Health Organization (WHO) have confirmed the veracity of the claimed human rights abuses. Republican Congressman Dana Rohrabacher describes how he successfully fought to have the issue brought to the attention of the U.S. Congress. The narrator asks: How can our society ignore these horrors? Is it compassion fatigue? Is it because of cultural or language dissonance? Is it because the public is unaware of the injustice suffered by prisoners and/or lack sympathy for their plight? A witness in the film recounts that one recipient accepted without question the information that his transplanted organ had come from a prisoner who had killed his family. The documentary moves into discussion about the idea that an issue may be so “difficult” as to make it publicly and politically untouchable. This is a proposed explanation of why this evil can be ignored. Rohrabacher suggests the idea that no-one wants to confront the Chinese on this sensitive topic because it may impact trade and foreign relations.

Ethicist Arthur Caplan expresses his disbelief that these practices are allowed to continue unchallenged, as he unpacks the litany of maleficence involved in the destruction of the principles that underpin legitimate organ donation. He specifically counts the absence of voluntary informed donor consent, the fact the “dead donor rule” is irrelevant and substituted with medical murder, the obfuscation of the organs’ provenance and distribution, and the removal of organs from executed prisoners as running counter to current standard of practice and as evidence of crimes against humanity. He argues that an absence of transparency allows these charges to be laid. Caplan asks, “Will we put up with it?” Gutmann informs us that an American best-selling book, *Larry’s Kidney*, which recounts a patient’s trip to purchase a kidney in China, is being made into a movie and that revealing and legitimizing the organ trade in this way will effectively advertise that China has organs for sale.

The film also argues that transparent practice and data trails can provide proof that organ donation is voluntary and informed—but evidence of such data trails for organ recovery, allocation, and transplantation is still lacking from China. The documentary presents the view that, to address this problem, China must display transparent evidence of adhering to the rules of legitimate organ donation—that donation is informed, voluntary, and transparently managed; that organs can only be taken after a person has died; that prisoners’ organs should never be used; that organs should never be taken from executed prisoners. Patients in need of a transplant should be fully informed of the risks of the organ trade, both to the donor and to themselves. Laws such as those initiated in Israel to prohibit patients from being covered by medical insurance if they purchase organs should be introduced in the United States and other countries where patients travel to purchase an illegal organ.

This documentary is extremely important for those involved in organ donation and transplantation, human rights, healthcare, ethics, and the law. A failure to address the needs of vulnerable people erodes humanity and destroys public trust. Politicians, policy–makers, and legislators can contribute to solving this problem. In the first instance, patients considering an organ purchase must be educated that their donor will be harmed and may die. Patients requiring transplantation need to realize that, regardless of their desperation, in purchasing an organ they are complicit in a crime. Rigorous efforts must be made to ensure each country maximizes its transplantation rate to meet the needs of its population using organ transplantation practices that align with World Health Organization principles. Please watch the documentary and come to your own conclusions.